# Expression of HMGCS2 in intestinal epithelial cells is downregulated in inflammatory bowel disease associated with endoplasmic reticulum stress

**DOI:** 10.3389/fimmu.2023.1185517

**Published:** 2023-06-30

**Authors:** Beatriz Martín-Adrados, Stefanie K. Wculek, Sergio Fernández-Bravo, Raúl Torres-Ruiz, Ana Valle-Noguera, Maria José Gomez-Sánchez, José Carlos Hernández-Walias, Frederico Moraes Ferreira, Ana María Corraliza, David Sancho, Vanesa Esteban, Sandra Rodriguez-Perales, Aránzazu Cruz-Adalia, Helder I. Nakaya, Azucena Salas, David Bernardo, Yolanda Campos-Martín, Elena Martínez-Zamorano, Diego Muñoz-López, Manuel Gómez del Moral, Francisco Javier Cubero, Richard S. Blumberg, Eduardo Martínez-Naves

**Affiliations:** ^1^ Department of Immunology, Ophthalmology and ORL, School of Medicine, Universidad Complutense of Madrid (UCM), Instituto de Investigación Sanitaria Hospital 12 de octubre (imas12), Madrid, Spain; ^2^ Immunobiology Laboratory, Centro Nacional de Investigaciones Cardiovasculares (CNIC), Madrid, Spain; ^3^ Department of Allergy and Immunology, IIS-Fundación Jiménez Díaz, Universidad Autónoma of Madrid, Madrid, Spain; ^4^ Molecular Cytogenetics & Genome Editing Unit, Human Cancer Genetics Program, Centro Nacional de Investigaciones Oncológicas (CNIO), Melchor Fernández Almagro, Madrid, Spain; ^5^ Centro de Investigaciones Energéticas Medioambientales y Tecnológicas (CIEMAT), Advanced Therapies Unit, Hematopoietic Innovative Therapies Division, Instituto de Investigación Sanitaria Fundación Jiménez Díaz, Universidad Autónoma de Madrid (IIS-FJD, UAM), Madrid, Spain; ^6^ LIM50, Division of Pathology, University of São Paulo School of Medicine, São Paulo, SP, Brazil; ^7^ Department of Gastroenterology, Instituto de Investigaciones Biomédicas August Pi i Sunyer (IDIBAPS), Hospital Clinic of Barcelona, Centro de Investigación Biomédica en Red-Enfermedades Hepáticas y Digestivas (CIBER-EHD), Barcelona, Spain; ^8^ Department of Clinical and Toxicological Analyses, School of Pharmaceutical Sciences, University of São Paulo (USP), Hospital Israelita Albert Einstein, São Paulo, SP, Brazil; ^9^ Gut Immunology Research Group, Instituto de Investigación del Hospital Universitario de la Princesa, Madrid, Spain; ^10^ Unidad de Excelencia Instituto de Biología y Genética Molecular (IBGM, Universidad de Valladolid-Consejo Superior de Investigaciones Científicas (CSIC)), Valladolid, Spain; ^11^ Centro de Investigaciones Biomédicas en Red de Enfermedades Infecciosas (CIBERINFEC), Instituto de Salud Carlos III, Madrid, Spain; ^12^ Department of Pathology, Hospital Universitario de Toledo, Toledo, Spain; ^13^ Department of Cellular Biology, School of Medicine, Universidad Complutense of Madrid (UCM), Madrid, Spain; ^14^ Centro de Investigaciones Biomédicas en Red de Enfermeddes Hepáticas y Digestivas (CIBEREHD), Barcelona, Spain; ^15^ Division of Gastroenterology, Department of Medicine, Brigham and Women’s Hospital, Harvard Medical School, Boston, MA, United States

**Keywords:** inflammatory bowel disease, inflammation, ER stress, HMGCS2, unfolded protein response (UPR)

## Abstract

**Introduction:**

The Unfolded Protein Response, a mechanism triggered by the cell in response to Endoplasmic reticulum stress, is linked to inflammatory responses. Our aim was to identify novel Unfolded Protein Response-mechanisms that might be involved in triggering or perpetuating the inflammatory response carried out by the Intestinal Epithelial Cells in the context of Inflammatory Bowel Disease.

**Methods:**

We analyzed the transcriptional profile of human Intestinal Epithelial Cell lines treated with an Endoplasmic Reticulum stress inducer (thapsigargin) and/or proinflammatory stimuli. Several genes were further analyzed in colonic biopsies from Ulcerative Colitis patients and healthy controls. Lastly, we generated Caco-2 cells lacking HMGCS2 by CRISPR Cas-9 and analyzed the functional implications of its absence in Intestinal Epithelial Cells.

**Results:**

Exposure to a TLR ligand after thapsigargin treatment resulted in a powerful synergistic modulation of gene expression, which led us to identify new genes and pathways that could be involved in inflammatory responses linked to the Unfolded Protein Response. Key differentially expressed genes in the array also exhibited transcriptional alterations in colonic biopsies from active Ulcerative Colitis patients, including NKG2D ligands and the enzyme HMGCS2. Moreover, functional studies showed altered metabolic responses and epithelial barrier integrity in HMGCS2 deficient cell lines.

**Conclusion:**

We have identified new genes and pathways that are regulated by the Unfolded Protein Response in the context of Inflammatory Bowel Disease including *HMGCS2*, a gene involved in the metabolism of Short Chain Fatty Acids that may have an important role in intestinal inflammation linked to Endoplasmic Reticulum stress and the resolution of the epithelial damage.

## Introduction

Inflammatory Bowel disease (IBD), including Crohn’s disease (CD) and Ulcerative Colitis (UC), is characterized by a chronic relapsing intestinal inflammation. Although its etiology remains unknown, IBD has been associated with alterations in the intestinal microbiota, failures in the control of the immune response as well as dysfunctions in the epithelial barrier (1). Intestinal Epithelial Cells (IECs) provide the first line of immune defense in the intestinal mucosa and respond to inflammatory signals by secreting large amounts of proteins, which make them prone to suffer Endoplasmic Reticulum (ER) stress and consequently highly dependent on the Unfolded Protein Response (UPR) (2).

ER stress is a condition characterized by the accumulation of misfolded or unfolded proteins within the ER lumen, to which the cell responds through the activation of the UPR.

In normal conditions, the UPR mediators ER transmembrane inositol-requiring enzyme 1α and 1β (IRE1α and β), the PRKR-Like Endoplasmic Reticulum Kinase (PERK) and the activating transcription factor 6 (ATF6) remain inactive and bound to the chaperone immunoglobulin-heavy-chain-binding protein (BIP), encoded by the *HSPA5* gene. In ER stress conditions the activation of the UPR takes place and BIP is released (3). Perturbations in the physiological levels of the UPR in IECs can lead to several inflammatory disorders, including IBD (4).

There is substantial evidence from mouse models (5, 6) and patient data (7, 8) demonstrating an association of genetic variants in ER stress genes and increased susceptibility to intestinal inflammation. Furthermore, in patients with IBD, there is activation of the UPR both in the mucosa of affected and unaffected disease areas (9, 10) and ER stress appears located mainly in the epithelial lining of the mucosa (11). ER stress and inflammation are closely intertwined, as inflammation can produce ER stress and conversely ER stress can initiate or enhance the inflammatory response, however the underlying epithelial cell intrinsic mechanisms behind the pathogenesis of IBD and, particularly, between ER stress and inflammation remain poorly understood. Therefore, we set out to identify novel UPR-activated mechanisms that might be involved in triggering or perpetuating the inflammatory response carried out by the IECs in the context of IBD.

Our results show that the inflammatory response in IECs is dramatically amplified in the presence of ER stress. We identified new genes and pathways regulated by the UPR in IECs, including HMGCS2, the rate-limiting enzyme of ketogenesis that may be crucial in IBD pathogenesis.

## Methods

### Cell culture

The human colorectal adenocarcinoma cell lines Caco-2 and HT-29 were grown in DMEM supplemented with 20% heat-inactivated fetal calf serum (FCS) and RPMI supplemented with 10% FCS respectively, 5% L-glutamine, 5% antibiotic-antifungal (Lonza, Basel, Switzerland) and 5% non-essential amino acids (Thermo Fisher Scientific, Walthman, CA USA). Both cell lines were cultured until reaching 70-80% confluence.

### Reagents and *in vitro* cellular treatments

Purified flagellin (SRP8029) was used at a final concentration of 1 μg/mL for 2 hours. Tunicamycin (TM) (T7765) at 2 μg/ml and thapsigargin (TG) (T9033) at 1 μM, for 5 or 7 hours. Seahorse experiments were performed with 98,5% pure Sodium butyrate. PERK-specific inhibitor GSK2656157 was used at 5 μM, IRE1α-specific inhibitor 4μ8c at 40 μM, and ATF6-specific inhibitor PF-429242 at 10 μM, for two hours prior to the stimulation with TG for 24 hours. All these reagents were purchased from Merck, Darmstadt, Germany.

### Total RNA extraction

RNA was extracted from cell lysates or whole tissues using the NucleoSpinRNA Mini Kit (Macherey-Nagel, Düren, Germany). For paraffined samples from Hospital Universitario de Toledo the kit miRNeasy FFPE Kit (217504) and Deparaffinization Solution (Qiagen, Venlo, Netherlands) were used.

### Microarray

Total RNA fraction from Caco-2 cells was analyzed using Human Gene Expression G3 v2 60K (Agilent microarray design ID 039494, G4851B) at the CNIO. The images background correction, quality assessment, normalization, statistical analysis and identification of differentially expressed genes (DEG) were performed using the R/Bioconductor LIMMA package. P-values were submitted to false discovery rate adjustment with the Benjamini-Hochberg method. Genes were considered differentially expressed if adjusted p ≤ 0.05 and absolute fold change ≥1.5 (12). Gene/Functional enrichment analysis were performed using the R/Bioconductor package fGSEA (13) and Ingenuity Pathway Analysis software. GO enrichment analysis was performed through ShinyGO v0.75 software.

### Transcript profiling

Expression microarray data are available at the NCBI GEO repository with GEO accession number GSE200626.

### Real time Q-PCR

Real time qPCR was performed in the Genomics Unit of C.A.I. from the UCM using ABI PRISM 7700(Applied Biosystems, Foster City, CA, USA). cDNA was done using High-Capacity cDNA Reverse Transcription Kit (Thermofisher, Thermo Fisher Scientific, Walthman, CA, USA). Primers were designed using the Universal Probe Library (UPL) software (Roche Diagnostics, Basel, Switzerland) (**Supplementary Table 1**).

### Patients

A total of 68 IBD patients admitted to the Hospital Universitario de La Princesa participated in the study, after providing written informed consent (25 biopsies were used to extract total RNA, 28 samples were used to isolate total protein and 15 samples were provided as paraffin embedded slides). cDNA samples from 17 patients provided by the Hospital Clinic of Barcelona and 18 patients provided by the BioB-HVS (B.0000520) were also included in the study. Biopsies were classified by a pathologist as non-IBD controls, quiescent IBD (in remission) or active IBD (with inflammation) and given a Mayo endoscopic sub- score. Non-IBD controls were those subjects undergoing colonoscopy for mild gastrointestinal symptoms or a screening for colorectal cancer, who presented no lesions during examination. This study was approved by the Research Ethics Committees from the Hospital Universitario de La Princesa, Hospital Clinic of Barcelona, Hospital Universitario de Toledo and Universidad Complutense de Madrid.

### Animal model of ER stress: intraperitoneal injection of tunicamycin

All animal procedures were conducted following the regulations of the European community and with the approval of the Research Ethics Committee from UCM. 11-week-old wild-type C57BL/6 mice (Charles River) were treated with an intraperitoneal injection of tunicamycin at 10 µg/gr mice and sacrificed at 24 hours. Colon was processed following the corresponding protocols. In addition, we treated in the same way *Xbp1* ^ΔIEC^ mice, which have a defective UPR due to a conditional deletion of *Xbp1* gene in IECs resulting in an ER-stress associated spontaneous small intestinal inflammation, but not colitis (5). C57BL/6 wild type and *Xbp1* ^ΔIEC^ mice were not littermates.

Animal procedures were approved by the Animal Care and Ethics Committee of the Universidad Complutense de Madrid and conducted following the sanitary recommendations of the Federation of European Laboratory Animal Science Associations (FELASA).

### Western blot

Total protein from cell cultures or colon samples were homogenized in RIPA buffer supplemented with Protease Inhibitor Cocktail (Thermo Fisher Scientific, Walthman, CA USA) and phosphatase inhibitors PhosSTOP™ (Roche, Basel Switzerland), resolved by SDS–PAGE and stained with the following primary (**Supplementary Table 2**) and secondary antibodies (**Supplementary Table 3**). Membranes were visualized using the Odyssey^®^ Fc Imaging System (LI-COR Biosciences).

### Immunohistochemistry

Slides of paraffined-tissue sections were provided by the Hospital Universitario de La Princesa. Retrieval of the antigen was achieved using Antigen retrieval basic (CTS013) (R&D Biosystems, Minneapolis, MN, USA) and stained using Anti-Rabbit HRP-DAB Cell & Tissue Staining Kit (CTS005) from R&D Biosystems with the primary antibodies in **Supplementary Table 4**.

### sgRNA design and lentiviral constructs generation

sgRNAs were designed and cloned as previously described (14). Briefly, sgRNAs were chosen based on a low off-target score and a high prediction rank for their specificity (Benchling CRISPR gRNA Design tool; http://www.benchling.com). Two different sgRNAs targeting the HMGCS2 gene (sgHMGCS2.1: AGCAGAAGCCCATACGGGTC and sgHMGCS2.2: GTTTGGTCCACATATTGGGC) and a control non-targeting guide (sgNT: CCGCGCCGTTAGGGAACGAG) were used.

### Lentivirus generation, titration, and transduction

Viruses were produced by transfection into HEK293T. Briefly, cells were seeded at 1.1 × 10^7^ cells/dish in 15-cm dishes and transfected using second-generation packaging plasmids (psPAX2 and pMD.2G, Addgene #12260 and #12259, respectively) and the appropriate transfer plasmid (pLV CRISPR sgHMGCS2.1_1, sgHMGCS2.2 or sgNT). The medium was collected after 48 hours, cleared by low-speed centrifugation, and filtered. Viral titers ranged around 10^7^ to 10^8^ TU/ml. Cells were transduced using a multiplicity of infection (MOI) of 5 and incubated at 37°C for 12 hours, and further analysis was performed by Raúl Torres at CNIO.

### Examination of mitochondrial respiratory function

The Seahorse XF-96 Extracellular Flux Analyzer (Agilent Technologies, Santa Clara, CA, USA) was used for determination of real-time oxygen consumption rate (OCR) and extracellular acidification rate (ECAR) using materials purchased from Agilent Technologies. 2.5x10^4^ Caco-2 cells were cultured for 24 hours in XF-96 Seahorse plates (Agilent Technologies Santa Clara, CA, USA), washed with PBS and the media changed to DMEM containing 100 μg/ml penicillin, 100 μg/ml streptomycin, phenol red and 25 mM glucose + 1mM pyruvate + 2mM glutamine (complete medium) or 5mM Sodium butyrate (butyrate medium) (Thermo Fisher Scientific, Walthman, CA USA). Three measurements were performed over 15 minutes in the basal condition, upon addition of 1μM oligomycin, 1μM FCCP, and 1μM rotenone + 1μM antimycin A (Thermo Fisher Scientific, Walthman, CA USA). The basal respiratory rate (BRR), maximal respiratory rate (MRR) and spare respiratory capacity (SRC) were calculated according to the manufacturer’s instructions.

### Cell viability assay

To address Caco-2 cell viability in response to different butyrate concentrations, MTT assay was performed. This assay is based on the reduction of a yellow tetrazolium salt (3-(4,5-dimethylthiazol-2-yl)-2,5-diphenyltetrazolium bromide or MTT) to purple formazan crystals by metabolically active cells.

To perform this experiment, Caco-2 control, Mock or HMGCS2-deficient cells were cultured 4 days prior to the experiment in complete low glucose medium: DMEM without glucose (11966025) from Gibco ™ supplemented with 20% heat-inactivated (60 min, 57°C) fetal calf serum (Lonza) and 1g/l glucose (G8270) from Sigma Aldrich. Cells were then seeded at 5000 cells/per 96 well in 100 µl of complete low glucose medium. Different concentrations of sodium butyrate were added 48 hours after the seeding in low glucose medium without serum. 48 hours after stimulation, the MTT protocol was performed following manufacturer’s instructions. MTT (M6494) from Life Technologies, S.A was added to the culture at a final concentration of 0.5 mg/ml in low glucose medium without serum and incubated for 3 hours at 37°C. The resulting formazan is then solubilized in 100 µl DMSO (D8418) from Sigma Aldrich, and the presence of formazan was determined by measuring the optical density at 570nm. The number of viable cells was addressed using the following formula:

% Viable cells = (Abs Samples - Abs Blank)/Abs control - Abs Blank) x 100 

### Determination of Trans-Epithelial Electrical Resistance (TEER).

Caco-2 cells were seeded at 5x10^4^ cells per well in 24-well Transwell^®^ dishes with porous filter membranes. Trans-Epithelial Electrical Resistance (TEER) of HMGCS2-deficient, Mock or control Caco-2 cells was measured using a Millicell-ERS system (Millipore, Burlington, MA, USA) every 48 hours and compared to the basal TEER levels to evaluate the integrity of the monolayers.

### Statistical analysis

Mean values were compared using the unpaired Student’s t-test, Mann Whitney or One-way ANOVA using GraphPad Prism 6.0 software. Statistically significant differences were represented as: *P<0.05, **P<0.01, ***P<0.001, ****P<0.0001. A p-value lower than 0.05 was considered statistically significant.

## Results

### The inflammatory response is dramatically amplified in IECs under ER stress

We designed an *in vitro* model which allowed us to study the pro-inflammatory response of IECs in the presence or absence of ER stress. To this end, we stimulated Caco-2 cells, an IEC cell line derived from a human colorectal adenocarcinoma, with the TLR5 agonist flagellin (Fl), with the chemical ER stress inducer thapsigargin (TG), or a combination of both (TGFl). This strategy mimics a pro-inflammatory response in the presence of ER stress. Treatment of the cells with TG efficiently activated the three UPR branches (**Supplementary Figure 1**). Next, we evaluated the transcriptional profile of Caco-2 cells in response to those treatment conditions through a Microarray-Based Gene Expression Analysis.

**Figure 1:**
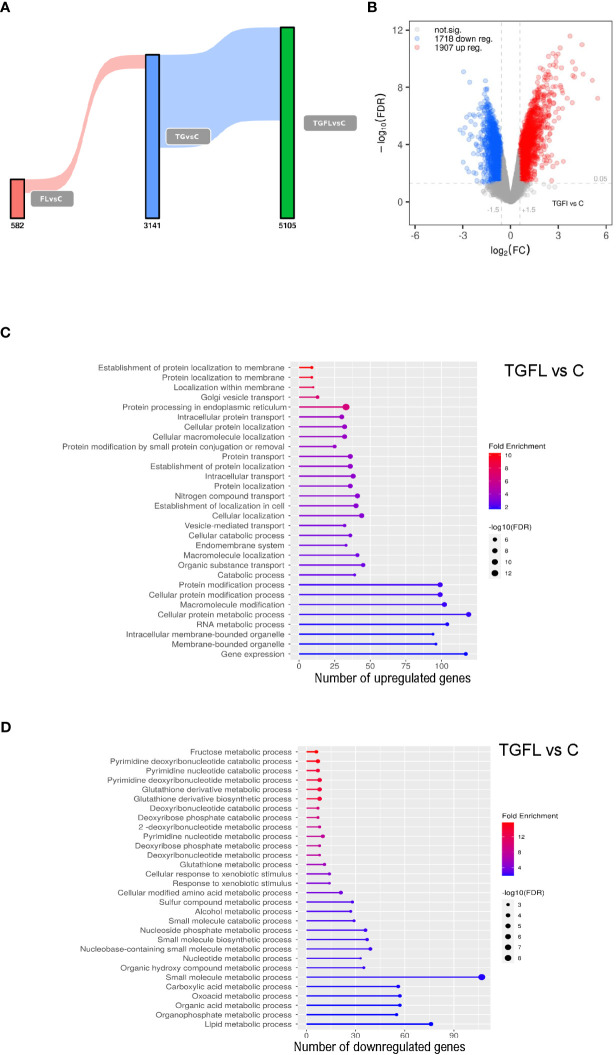
Transcriptional profile analysis of Caco-2 cell line upon inflammatory signals and ER stress induction. **(A)** Sankey plot representing all differentially expressed genes (DEGs) between groups, with a significance level of 0.01. Different treatments are represented as C (non-treated cells), Fl (treated with flagellin 1 μg/mL for 2 hours), TG (treated with TG at 1 μM for 5 hours), or TGFl (treated with 1 μM TG for 5 hours, and with 1 μg/mL Fl for additional 2 hours). Figures below the bars indicate the number of DEGs. **(B)** Volcano plot representing DEGs s between TGFl treatment and non-treated cells (control). The axis shows the logarithms of Fold Change (FC) and p-value. Black dots represent all the genes with significance level of < 0.05. **(C, D)** GO enrichment analysis representing the 30 top upregulated **(C)** and downregulated **(D)** pathways based on DEGs by biological process (BP). Selected genes were those upregulated **(C)** and downregulated **(D)** between TGFl and Cl treatments, using a p-value cut-off of 0.01. The color represents the Fold enrichment, the length of each bar represents the number of upregulated genes associated to that pathway and the size of the circle represents the False Discovery Rate (FDR).

Our data revealed a unique transcriptional signature associated with each treatment. Stimulation with flagellin altered the expression of 582 genes when compared to control cells. After TG treatment, we found alterations in the transcription of 3141 genes. Exposure to flagellin after thapsigargin treatment resulted in a powerful synergistic effect between the UPR and TLR signaling, altering the expression of 5105 genes (**Figures 1A, B**; **Supplementary Figures 2A–C**).

Using Ingenuity Pathway Analysis (IPA) we determined the most significant biological pathways altered in this *in vitro* model. A list of the top regulated genes and pathways in the different experimental settings is provided in **Supplementary Figure 3**. As expected, TLR5 stimulation altered proinflammatory pathways such as IL-6 and acute phase response gene signaling among others. ER stress induction via TG activated inflammatory cascades like IL-6 and IL-8 signaling, and interestingly, downregulated several metabolism-related pathways including glycolysis and oxidative phosphorylation suggesting mitochondrial dysfunction. Lastly, combined exposure to flagellin after thapsigargin treatment resulted in a powerful synergistic effect between ER Stress and TLR signaling, which resulted in dramatic quantitative and qualitative changes, that included the alteration of UPR pathways, several proinflammatory cascades like IL-6, IL-17 and NFKB, and general detoxification pathways (**Supplementary Figure 3**).

Supplementary GO enrichment analysis based on differentially expressed genes by biological process between the TGFl and C groups revealed upregulation of genes related to UPR, ER Stress and protein transport (**Figure 1C**), whereas the downregulated genes were mostly related to mitochondrial energy and various metabolic processes (**Figure 1D**).

**Figure 2:**
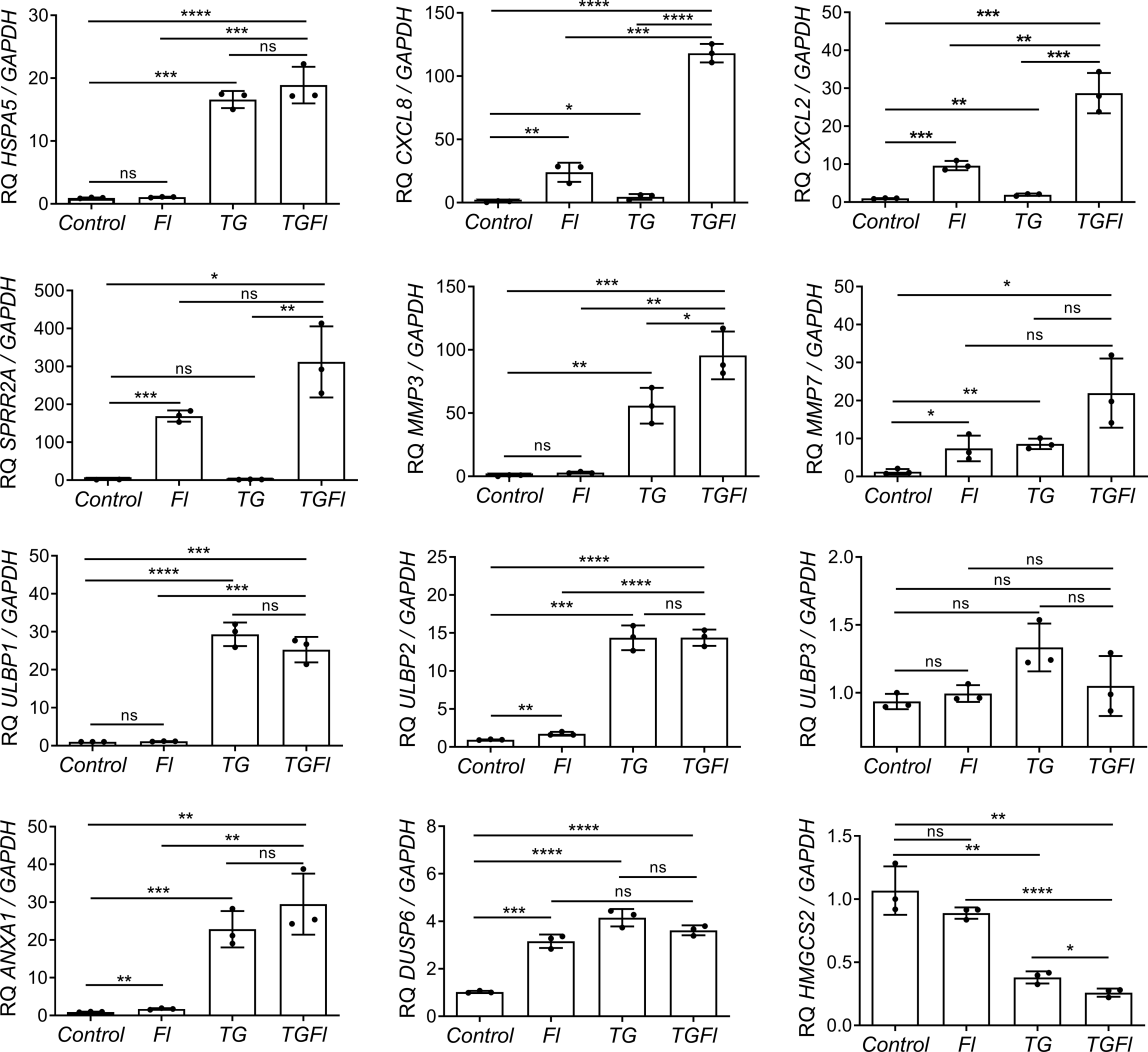
Changes in Caco-2 gene expression by flagellin (Fl), thapsigargin (TG) or the combination of both treatments (TGFl). Effect of indicated (Fl, TG or TGFl) treatments compared to non-treated cells (Control), on the expression of ER stress-related (*HSPA5*), chemokines and cytokines (*CXCL8, CXCL2*), epithelial reparation (*SPRR2A, MMP3, MMP7*), NKG2D ligands (*ULBP1, ULBP2, ULBP3*), cellular proliferation and differentiation (DUSP6, ANXA1) and mitochondrial (*HMGCS2*) genes. Caco-2 cells were incubated with flagellin 1 μg/mL for 2 hours, with 1 μM TG for 5 hours or with TG for 5 hours and Fl for additional 2 hours. The relative mRNA abundance of the indicated genes was determined by qPCR using GAPDH as the housekeeping gene. Data from three independent experiments are shown, represented as the mean ± SEM of individual groups compared with the control group. Unpaired t test was used. *P<0.05, **P<0.01, ***P<0.001, ****P<0.0001. ns: not significant.

We selected several genes for further validation by Q-PCR (**Figure 2**). Some genes like *CXCL2* and *SPPR2A*, displayed dramatic changes in their expression patterns specifically after treatment with flagellin. Other genes, such as *HMGCS2*, NKG2D ligands *ULBP1* and *ULBP2*, or *ANXA1*, were found to be solely regulated by ER stress (thapsigargin). Some genes like those encoding the metalloprotease *MMP7*, the chemokines *CXCL8* or *CXCL2*, were synergistically regulated by the combined treatment (TGFl). In contrast *DUSP6* was upregulated by both flagellin and thaspsigargin, but there was not synergistic effect by the combined treatment.

### Transcriptional alterations in intestinal biopsies from IBD patients phenocopy those induced by ER stress in IECs *in vitro*

**Figure 3:**
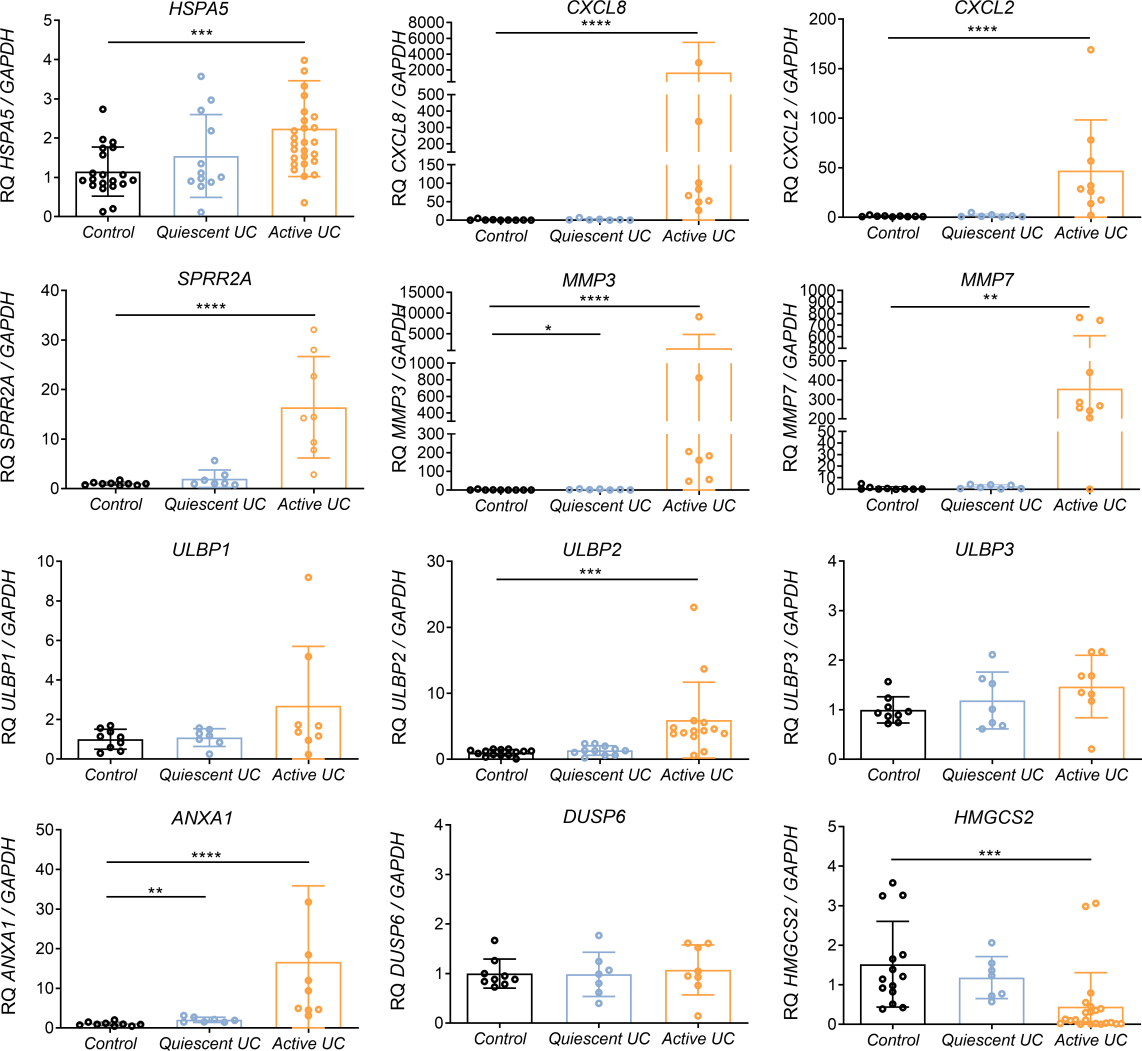
Relative mRNA expression of selected genes in colonic biopsies from UC patients and healthy controls. RNA colonic samples from active or quiescent UC patients and healthy controls was analyzed by qPCR. Relative mRNA expression levels of ER stress-related (*HSPA5*), chemokines and cytokines (*CXCL8, CXCL2*), epithelial reparation (*SPRR2A, MMP3, MMP7*), NKG2D ligand genes (*ULBP1, ULBP2, ULBP3*), cellular proliferation and differentiation (DUSP6, ANXA1) and mitochondrial (*HMGCS2*) genes is shown, using GAPDH as the housekeeping gene. Mann-Whitney U test was used. Data are represented as the mean ± SEM *P<0.05, **P<0.01, ***P<0.001, ****P<0.0001.

Those genes which were validated in IECs lines, were then transcriptionally analyzed in colonic biopsies from UC patientes with active or quiescent disease and compared to healthy controls. Interestingly, key differentially expressed genes in the array also exhibited transcriptional alterations in colonic biopsies from active UC patients (**Figure 3**). As expected, *HSPA5* and *CXCL8* were found to be upregulated (**Figure 3**) in those patients with active disease. Moreover, the gene encoding *CXCL2*, and genes related to tissue reparation as *SPRR2A*, *ANXA1*, *MMP3* and *MMP7*, were also strongly upregulated in this same group of patients (**Figure 3**). The mRNA of *ULBP1* and *ULBP2* genes but not that of *ULBP3* was increased in the colonic mucosa of patients with active UC (**Figure 3**), interestingly, the same was observed in the experiments with Caco-2 cells treated with TG (**Figure 2**). In contrast DUSP6 was not upregulated in the mucosa of IBD patients although it was upregulated by flagellin and thapsigargin “*in vitro*” (**Figure 2**). Lastly, *HMGCS2* expression was strongly downregulated in patients with active but not quiescent disease (**Figure 3**).

### HMGCS2 expression is decreased in colonic samples from patients with active IBD

Among all the genes analyzed, we decided to focus on those whose transcription is reduced under ER stress, since protein expression is also attenuated by the UPR (15) and would therefore have more drastic functional effects. Several mitochondrial pathways were among the most downregulated by ER stress (**Figure 1D**), including HMGCS2, which we considered to be of particular interest in IBD for several reasons. *HMGCS2* encodes the mitochondrial protein 3-Hydroxy-3-Methylglutaryl-CoA Synthase 2 which is the rate-limiting enzyme in ketogenesis, a metabolic pathway that provides lipid-derived energy for various organs at moments of carbohydrate deprivation. HMGCS2 is mainly expressed in the liver and gastrointestinal tract, particularly in apical colonocytes whose main energy source is the butyrate (16) produced by the commensal microbiota. Inside the colonocytes butyrate is converted to acetyl-CoA through β-oxidation prior to energy production in the TCA cycle and electron transport chain. Acetyl-CoA is also the substrate for ketogenesis which produces beta-hydroxybutyrate. In situations of excess acetyl-CoA (TCA cycle metabolic capability is exceeded) or lack of TCA cycle intermediates, acetyl-CoA is increasingly converted to ketone bodies such as beta-hydroxybutyrate that can be converted back to acetyl-CoA to be used for energy production. In this way ketone bodies provide an alternative fuel that is augmented when increases fatty acid availability and diminishes carbohydrate availability. Moreover, ketosis and ketone bodies modulate inflammation and the immune response, and beta-hydroxybutyrate has been shown to have several anti-inflammatory effects (17). In addition, HMGCS*2* has recently been described to be expressed in intestinal epithelial stem cells, where it is a regulator of their self-renewal and differentiation into different lineages (18, 19).

Thus, we decided to study in depth the relationship between the UPR and HMGCS2 expression. Firstly, we examined whether HMGCS2 could be downregulated by other ER stress mediators, such as tunicamycin (TM). We found that both chemical ER stress inducers (TG and TM) which effectively activated the UPR as shown by *HSPA5* induction, strongly decreased *HMGCS2* transcription in two different human colorectal cell lines, Caco-2 and HT29 (**Supplementary Figure 4**).

To evaluate the possible involvement of HMGCS2 in human IBD, we further studied the expression pattern of HMGCS2 in colonic biopsies of UC patients and healthy controls. As shown in **Figure 3**, our results confirmed a downmodulation or complete loss of HMGCS2 expression in patients with active UC, regardless of the treatment (**Supplementary Figure 5A**), or their severity score (**Supplementary Figure 5B**), when compared to healthy controls. HMGCS2 expression in patients with quiescent disease was comparable to that of healthy controls.

**Figure 4:**
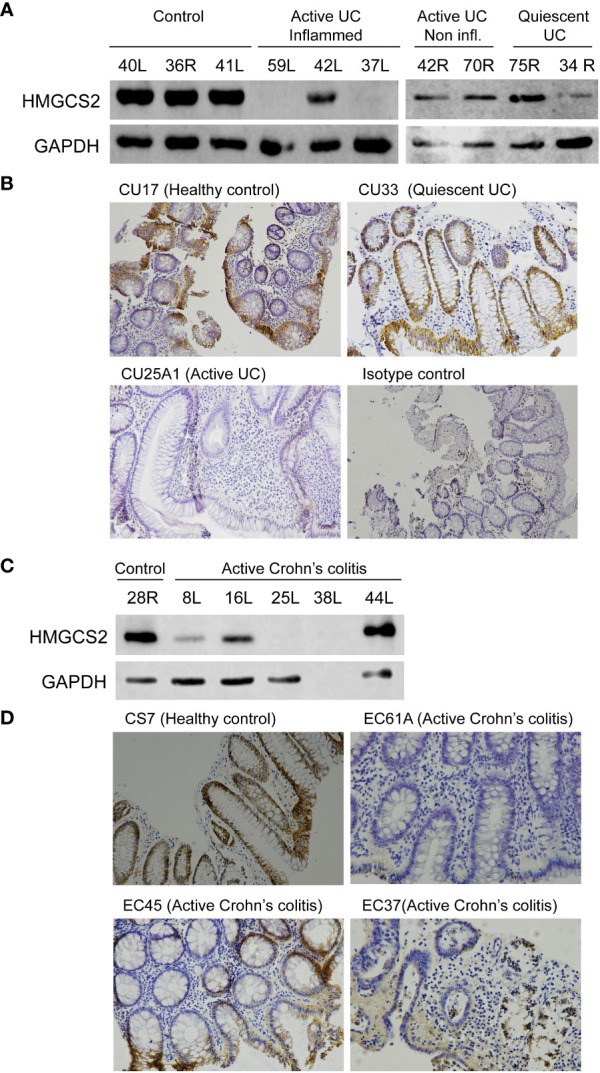
HMGCS2 protein expression is decreased in colonic samples from IBD patients. **(A)** Representative immunoblot of HMGCS2 expression in colonic samples from UC patients with active or quiescent disease or healthy controls compared to GAPDH expression. **(B)** Representative HMGCS2 immunohistochemistry using paraffined-tissue slides. Samples CU17 (Healthy control) and CU33 (Quiescent UC) had positive staining for HMGCS2, whereas CU25A1 (Active UC) displayed no staining for HMGCS2. Sample CU33 (Isotype control) was used as a negative control. **(C)** Representative immunoblot of HMGCS2 expression in colonic samples from healthy controls and patients with active colonic Crohn’s disease. **(D)** Representative HMGCS2 immunohistochemistry of active Crohn’s colitis biopsies (EC61A and EC37) which showed no staining, whereas healthy control biopsy (CS7) and active Crohn’s colitis biopsy (EC45) were positive for HMGCS2 staining.

We then investigated if this modulation occurred also at protein level using western blot and immunohistochemistry techniques (**Figures 4A, B**). We found that 5 out of the 7 patients with active UC displayed a dramatic reduction of HMGCS2 expression in the colon, whereas in samples obtained from patients in remission (0 out of 5) or healthy controls (0 out of 10) the protein was always detectable at high levels.

To investigate whether the downregulation of HMGCS2 is specific to UC or concerns IBD globally, we analyzed the expression of this enzyme in colonic tissue samples of active Crohn’s colitis patients and healthy controls. Consistently, the protein was absent or attenuated in the intestinal epithelia of 3 out of 4 (**Figure 4C**) as observed by western blot and of 2 out of 3 (**Figure 4D**) as determined by immunohistochemistry patients with active Crohn’s colitis disease. Together, these findings prove that HMGCS2 expression is dramatically reduced in colonic samples from patients with active IBD, suggesting that this enzyme may have a role in this pathology.

### *HMGCS2* transcription is modulated by ER stress via PERK signaling in Caco-2 cells

**Figure 5:**
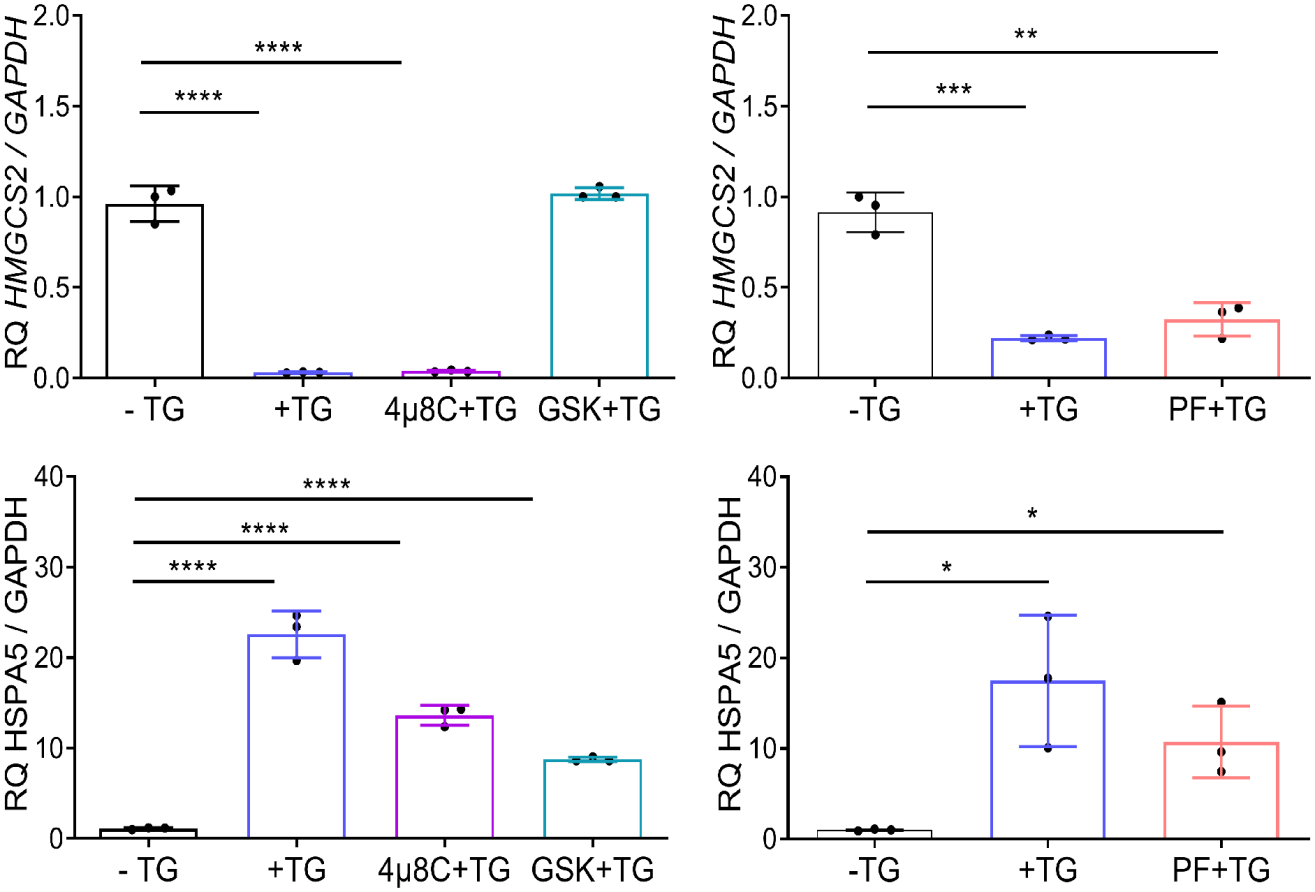
HMGCS2 is downregulated by ER stress through the activity of PERK. *HMGCS2* (upper graphs) or *HSPA5* (lower graphs) expression by qPCR in non-treated Caco-2 cells (-TG), cells treated with TG 1μM alone for 24 hours (+TG) or Caco-2 cells treated with pharmacological inhibitors of the three branches of the UPR and TG, using GAPDH as the housekeeping gene. Cells were pretreated with each inhibitor (GSK, 4µ8C or PF) for 2 hours and then stimulated with TG 1μM for additional 24 hours. Data from three independent experiments are shown, represented as the mean ± SEM. Unpaired t test was used. *P<0.05, **P<0.01, ***P<0.001, ****P<0.0001.

To identify the molecular mediators of *HMGCS2* downregulation in Caco-2 cell line, we used pharmacological inhibitors of the three branches of the UPR. We induced ER stress through addition of TG in the presence of GSK2656157 (GSK), 4µ8C and PF-429242 (PF), chemical inhibitors of PERK, IRE1 and ATF6 respectively. As seen in **Figure 5** *HMGCS2* transcription was similar in cells treated with TG alone, TG plus 4µ8C or TG plus PF. In contrast, cells treated with TG plus PERK inhibitor GSK showed HMGCS2 levels comparable to those of control cells. This indicates that *HMGCS2* is downregulated by ER stress in Caco-2 cells by a mechanism dependent on the activity of PERK.

### ER stress is sufficient to induce HMGCS2 downregulation *in vivo*

Our results show that a majority of IBD patients with active disease display a dramatically reduced expression of this enzyme in their colon.

**Figure 6:**
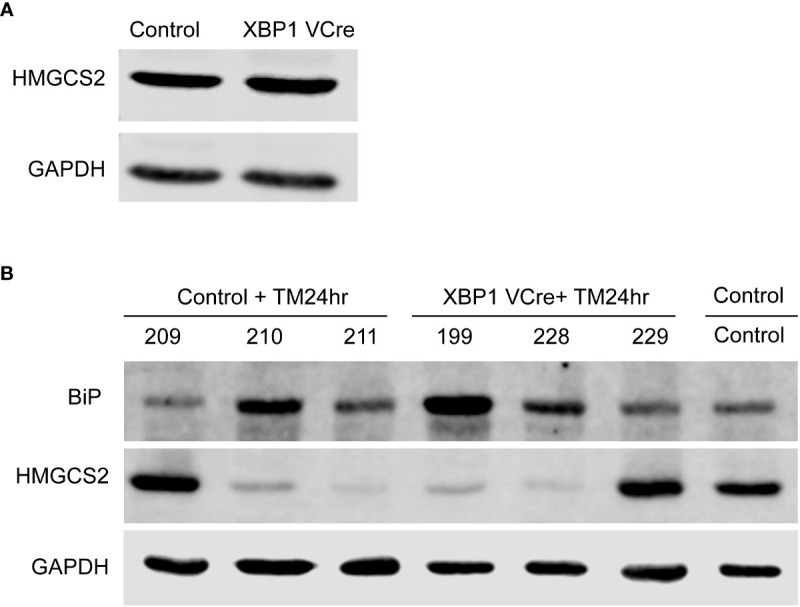
Chemical ER stress induces HMGCS2 downregulation in the colon of C57BL/6 mice. **(A)** Representative immunoblot showing that HMGCS2 expression in the colon of *Xbp1* ^ΔIEC^ mice is equivalent to that of C57BL/6 WT mice, normalized to GAPDH expression. **(B)** Immunoblot of colonic mucosal samples from mock C57BL/6 WT mice (Control) and C57BL/6 WT (Control+TM24hr) or XBP1 Vcre (XBP1 Vcre+TM24hr) mice after treatment with 10 µg/gr intraperitoneal tunicamycin for 24 hours. BIP stainings normalized to GAPDH show an activated UPR in tunicamycin injected mice, which correlates with diminished expression of HMGCS2. Each number represent a different mouse.

To investigate if the UPR could directly induce HMGCS2 downregulation *in vivo*, we injected tunicamycin in C57BL/6 wild type mice as well as in *Xbp1* ^ΔIEC^ mice, which display a defective UPR through the deletion of *Xbp1* in IECs resulting in an ER associated spontaneous enteritis, but not colitis (5). Untreated WT and *Xbp1* ^ΔIEC^ mice exhibited similar HMGCS2 levels in the colonic mucosa (**Figure 6A**). 24 hours after tunicamycin treatment, both WT and *Xbp1* ^ΔIEC^ animals had an activated colonic UPR as shown by a dramatic increase in the expression of BIP, which correlated with diminished expression of HMGCS2 (**Figure 6B**). Taken together these results strongly suggest that HMGCS2 is downregulated by the activation of UPR both *in vitro* and *in vivo*.

### Lack of HMGCS2 expression in Caco-2 alters epithelial cell functionality

**Figure 7:**
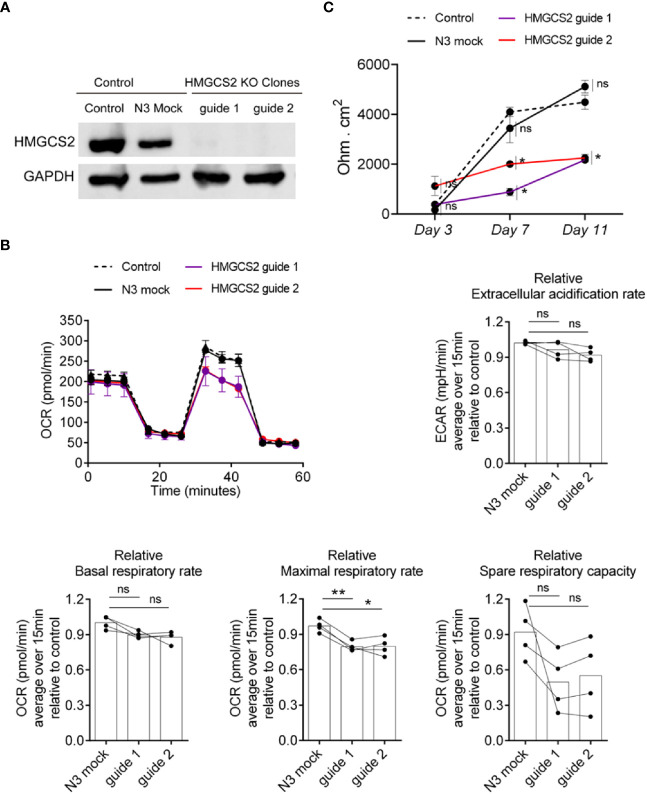
HMGCS2 absence associates with functional alterations in Caco-2 cells. **(A)** CRISPR/Cas9 knockout of HMGCS2. Immunoblot showing HMGCS2 protein expression in parental cells (Control), Mock cells (N3) and two different clones of HMGCS2 knock-out cells (guide 1 and guide 2 respectively), normalized to GAPDH expression. **(B)** HMGCS2 knock-out cell lines have a deficient Maximal Respiratory Rate. Bioenergetic analyses of Control Caco-2 (control parental cells and Mock Caco-2) and HMGCS2-deficient Caco-2 cell lines in medium with 5mM butyrate in response to different metabolic inhibitors. The results are normalized to control parental Caco-2 cell values. Oxigen consumption rates (OCR) and Extracellular acidification rate (ECAR) were measured three times over 15 minutes as a readout for the mitochondrial respiration and glycolysis respectively. Data shows a representative respiration plot (up left) or the mean and paired individual data points of 4 independent experiments. One-Way ANOVA corrected with Tukey’s multiple comparisons test was used. ns not significant (P>0.05), *P<0.05 **P<0.01. **(C)** Knockout of HMGCS2 increases the permeability of the intestinal monolayer. Control Caco-2 (control parental cells and Mock Caco-2) and HMGCS2-deficient Caco-2 cell lines at steady-state (basal) were seeded at the same concentration using complete DMEM medium. TEER was measured at the indicated time points and is given as raw TEER measurements normalized to wells without cells. Data represents the mean ± SEM of three independent experiments. One-way ANOVA corrected with Holm-Sidak’s multiple comparisons test was used. ns not significant (P>0.05), *P<0.05.

The alteration found in colonic samples from active IBD patients raised the question of whether the absence of HMGCS2 could be compromising intestinal epithelial cells by altering key biological functions. To address this, we knocked out HMGCS2 in Caco-2 cells (**Figure 7A**) using CRISPR Cas9 technique, generating two clones deficient in HMGCS2 by targeting different regions of the gene.

It has been reported that butyrate plays a concentration dependent role in the cell, promoting apoptosis when its intracellular amount increases (20). We therefore decided to analyze the viability of wild type Caco-2 and HMGCS2 KO cells in the presence of different butyrate concentrations using MTT assays. Our results indicate that Caco-2 cells undergo apoptosis in a butyrate-dependent manner, but we observed no significant differences between wild-type and HMGCS2 KO cells (**Supplementary Figure 7A**). We then decided to perform metabolic studies of these cells in the presence or absence of butyrate. To this end we measured the oxygen consumption rates (OCR) and the extracellular acidification rates (ECAR) as a readout of the mitochondrial respiration and the glycolytic metabolism, respectively. HMGCS2-deficient (HMGCS2-KO) and control cell lines (control parental and Mock Caco-2 cell lines) were cultured in glucose, glutamine and pyruvate-containing DMEM medium without butyrate (complete medium), or glucose-free DMEM medium with 5mM butyrate (butyrate medium). When cultured in complete medium, all cell lines displayed largely similar respiration parameters (**Supplementary Figure 7**). However, when cultured in butyrate medium (without glucose), HMGCS2-deficient cell lines showed a consistent reduction in their maximal respiratory rates (**Figure 7B**). Moreover, a tendency to a lower spare respiratory capacity was observed in those cells lacking HMGCS2. Taken together these results demonstrate that the lack of HMGCS2 affects the ability of IECs to respond to an energetic demand under stressful situations.

To further analyze the biological function of HMGCS2 in IECs, we evaluated the integrity of the physical barrier they could form by measuring the transepithelial electrical resistance (TEER). As expected, control cell lines displayed increased TEER values as they differentiate. Interestingly, HMGCS2-deficient cells lines showed reduced values for TEER measurements at days 7 and 11 when compared to their control counterparts (**Figure 7C**). This result proves that the integrity of the epithelial barrier formed by these cells is significantly reduced, indicating that cells lacking HMGCS2 are functionally impaired.

## Discussion

Our microarray results revealed that the addition of a proinflammatory stimulus to IECs undergoing ER stress synergistically potentiates the inflammatory response and altered additional pathways such as general detoxification pathways. Individual genes that we validated included those encoding proteins involved in tissue reparation (*MMP3*, *MMP7*), cellular proliferation and differentiation (*ANXA1, DUSP6*) as well as cytokines and chemokines (*CXCL8*, *CXCL2*). We also found alterations in NKG2D ligands (*ULBP1*, *ULBP2*) and mitochondrial genes (*HMGCS2*). These observations extend to human IBD, as *in vitro* results showed a strong correlation with our findings in colonic samples from patients.

We have previously shown that ER stress induces NKG2D ligand expression in murine intestinal epithelial cells resulting in the development of spontaneous enteritis in *Xbp1* ^ΔIEC^ mice (21). Moreover, expression of NKG2D ligands have been found in the intestinal epithelium of Crohn’s disease patients (22). In the present study, we prove that enhanced expression of NKG2D ligands also takes place in human colonic cells lines under ER stress conditions and in human clinical IBD samples, suggesting that they could have an important role in the pathogenesis of this disorder. Interestingly, a recent clinical trial showed improvement in some CD patients after treatment with an antibody against NKG2D (23). ANXA1 plays an important role as a regulator of the inflammatory process (24). It was upregulated by ER stress in Caco-2 cells in our experiments and its expression was also increased in the mucosa of UC patients with active disease, probably reflecting the presence of a compensatory mechanism to quench inflammation. DUSP6 has been reported to regulate colonic inflammatory responses and to protect the intestinal epithelium against oncogenic stress (25). Our results showed that DUSP6 was upregulated by both the UPR and flagellin *in vitro*, but we did not observe changes in expression between patients and healthy individuals, which may illustrate the complexity of the inflammatory response in IBD. This was the only gene where we did not observe a correlation between *in vitro* and patient results.

Expression of mitochondrial genes, like *HMGCS2* were also found altered in our microarray analysis of IECs receiving proinflammatory signals while experiencing ER stress. HMGCS2 is crucial to metabolize butyrate, the main energy source for IECs (16), and has very recently been found to be downregulated in the colon of patients with IBD (26–28). Here, we provide additional evidence to support these findings. Moreover, we prove that HMGCS2 repression only takes place in those patients undergoing inflammation but does not occur in patients with quiescent disease. Our results strongly suggest that ER stress is the main mechanism involved in HMGCS2 downregulation. Inflammation “per se” is very unlikely to be responsible since we found normal levels of HMGCS2 expression in the mucosa of some patients with high inflammatory activity. Furthermore, in a model of DSS-induced colitis the mice displayed increased levels of HMGCS2 (29). HMGCS2 expression is dependent on its substrate, butyrate (30), and IBD patients generally have a reduction in intestinal butyrate-producing bacteria (31), which could explain the lack of HMGCS2 in the inflamed gut. In contrast, our results show that chemically induced ER stress was sufficient to drastically reduce HMGCS2 expression *in vitro* as well as *in vivo*. Tunicamycin-injected mice showed a decrease in HMGCS2 expression, which correlated with an increase in BiP expression. In addition to wild-type mice, we decided to use *Xbp1* ^ΔIEC^ mice because they are a model of small intestinal inflammation associated with ER stress. In these mice, we found that untreated mice have normal levels of HMGCS2, but after tunicamycin treatment, HMGCS expression decreased to a level comparable to wild type. These data suggest that the decrease in HMGCS2 expression in mice is independent of the IRE1α-XBP1 pathway and may be mediated by a PERK-dependent mechanism, as in human cells.

Moreover, using specific inhibitors of the three UPR pathways, we showed that PERK was responsible for *HMGCS2* modulation in IEC lines. *HMGCS2* loss in intestinal stem cells skews their differentiation towards secretory cell phenotypes (18). Interestingly, the PERK-mediated UPR has been implicated in the protection of mitochondria and the promotion of mitochondrial metabolism (32). thus, the UPR- could well be an evolutionary mechanism aimed at fostering intestinal epithelial cell protection and renewal.

In the gut, IECs form a physical barrier whose dysfunction has been associated with an increasing variety of diseases, including IBD*. HMGCS2* knockdown attenuates spontaneous differentiation of Caco-2 cells into completely functional phenotypes, suggesting that their susceptibility to inflammation could be increased (18, 19). Thus, we hypothesized that HMGCS2 deficiency in our KO Caco-2 cells would lead to poorly differentiated enterocytes resulting in functionally impaired cells, compromising their barrier function. Consistent with this, our TEER data provided evidence that the absence of HMGCS2 perturbs the integrity of the epithelial monolayer. This could well be due to the attenuation of the differentiation process that KO Caco-2 cells undergo when cultured on transwell dishes. This is consistent with the fact that the differences between the wild type and the KO cells in TEER measurements are not significant at day 3, but they are significant at days 7 and 11, when Caco-2 would be more differentiated.

Butyrate plays a concentration dependent role in the cell, promoting apoptosis when its intracellular amount increases. Indeed, this is what we observed in our cell viability assays in the presence of increasing concentrations of butyrate. At low concentrations (between 0.01 and 1mM butyrate) there is an increase in viability, which is consistent with previously published (20) data indicating that at these concentrations, butyrate induces cell proliferation. In contrast, at 5 mM and above, the induction of apoptosis is very clear, with KO cells apparently being as sensitive as wild-type cells. This could be because cells KO for HMGCS2 were less efficient at taking up butyrate or were able to metabolize it in the TCA cycle after conversion to acetyl-CoA. It may also be that butyrate at very high concentration cannot be metabolized efficiently enough even in cells with normal HMGCS2 levels,

Our metabolic data using HMGCS2 deficient cell lines suggested that indeed, these cells displayed pronounced alterations in mitochondrial respiration only in the absence of glucose and the presence of butyrate. In this case, since HMGCS2 is the limiting enzyme of ketogenesis, KO cells would have no ketone bodies (beta-hydroxybutyrate) available (33) to support their maximal mitochondrial respiration when TCA cycle is saturated. On the other hand, it is possible that reduced mitochondrial acetyl-CoA metabolism in the absence of ketogenesis leads to excess acetyl-CoA, which, through its conversion to malonyl-CoA by the action of acetyl-CoA carboxylase, could inhibit beta-oxidation of butyrate (34). In turn, less acetyl-CoA may be available during situations requiring maximal respiration.

This situation would mainly occur in apical colonocytes, which express the highest levels of HMGCS2 under normal physiological conditions. In addition to metabolic inefficiency in these apical cells, lack of HMGCS2 can lead to butyrate accumulation in crypts, where it has been shown to inhibit intestinal stem cell proliferation (35).

Different groups have focused on studying the potential therapeutic effects of butyrate administration to treat UC. However, neither butyrate enemas (36–39) nor oral administration (40, 41), reported conclusive results although variable degrees of improvement were achieved in some UC patients (42). Based on our findings, we hypothesize that the subset of patients with active disease that do not express HMGCS2 in the colon could be representing the fraction of “non-responding” patients that systematically appeared in all the inconclusive trials. Stratification of patients according to colonic HMGCS2 expression levels might indeed identify patient groups with the potential to benefit from butyrate supplementation.

Altogether, we have shown that ER stress induces the downregulation of HMGCS2 in IEC lines, in the colon of mice with ER stressed colonocytes, and in colonic samples from a subset of patients with active IBD. The mechanisms involved in IBD pathophysiology are still not resolved, nor if *HMGCS2* modulation is a cause or consequence of the disease. However, we may have provided a logical hypothesis for the empirical failure of butyrate administration in previous trials. Our data point to a crucial role of HMGCS2, whose expression allows us to discern between two groups of patients, potentially generating a new approach in the development of new therapeutic strategies to improve this disease.

## Conclusions

We have discovered new pathways and genes activated by inflammatory stimuli associated with ER stress “*in vitro*”. The “*in vitro*” findings have been validated in biopsy samples from IBD patients. The expression of HMGCS2, the ketogenesis-limiting enzyme is absent in the inflamed tissue of most IBD patients. HMGCS2 expression is repressed by the Unfolded Protein Response in intestinal epithelial cells. The absence of this enzyme in Caco-2 cells is associated with increased permeability of the intestinal epithelial barrier and with metabolic alterations that would limit the response of these cells in situations of energy demand.

## Data availability statement

The datasets presented in this study can be found in online repositories. The names of the repository/repositories and accession number(s) can be found in the article.

## Ethics statement

The studies involving human participants were reviewed and approved by Research Ethics Committees from the Hospital Universitario de La Princesa, Hospital Clinic of Barcelona, Hospital Universitario de Toledo and Universidad Complutense de Madrid. The patients/participants provided their written informed consent to participate in this study. The animal study was reviewed and approved by Animal Care and Ethics Committee of the Universidad Complutense de Madrid.

## Author contributions

EM-N devised the project, designed experiments, and interpreted data. BM-A performed, designed, and interpreted most *in vivo* and *in vitro* experiments. SW performed, interpreted, and analyzed Seahorse experiments with the help of DS. SF-B and BM-A performed, interpreted, and analyzed TEER experiments with the help of VE. RT-R designed and provided the HMGCS2 CRISPR Cas9 lentiviral constructs with the help of SR-P. AV-N and MG-S performed, interpreted, and analyzed *C. rodentium* experiments with the help of AC-A. JH-W performed the microarray experiment. FF analyzed the microarray experiment with the help of HN. FJC performed IPA analysis of the microarray. AC, AS, DB, YC-M, EM-Z and DM-L provided patient samples. SW, RT-R, FF, AS, YC-M, MGM, FJC and RB contributed important intellectual insights and critically reviewed the manuscript. BM-A and EM-N wrote the manuscript with the help of all other authors. All authors contributed to the article and approved the submitted version.
