# Supplementary material for: Expression of HMGCS2 in intestinal epithelial cells is downregulated in inflammatory bowel disease associated with endoplasmic reticulum stress
Source: Front Immunol. 2023 Jun 30;14:1185517. doi: 10.3389/fimmu.2023.1185517 (PMC10348483; doi:10.3389/fimmu.2023.1185517)
Supplement: Supplementary file 1 [file DataSheet_1.pdf]

Supplementary Figure 1

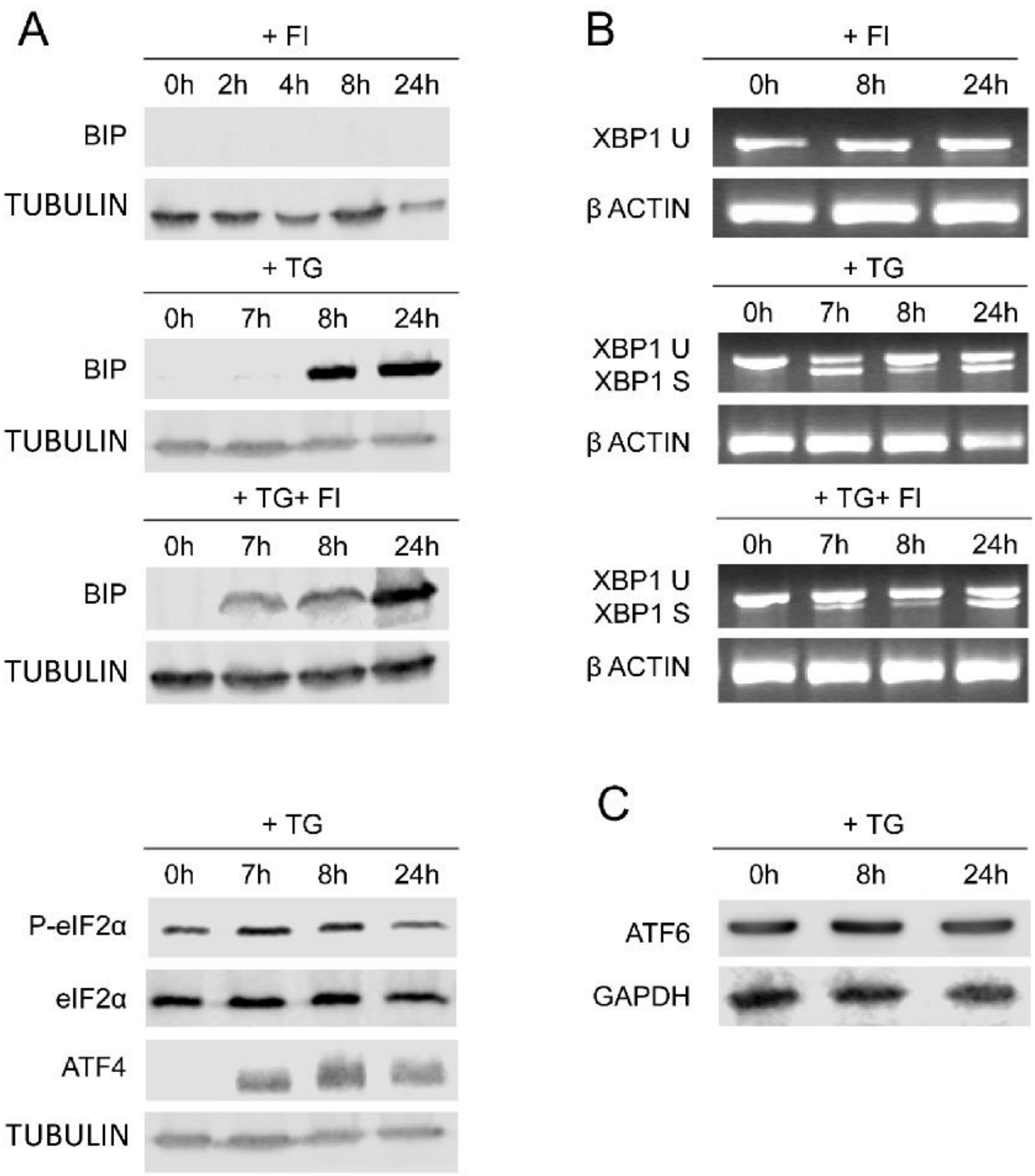

Supplementary Figure 2

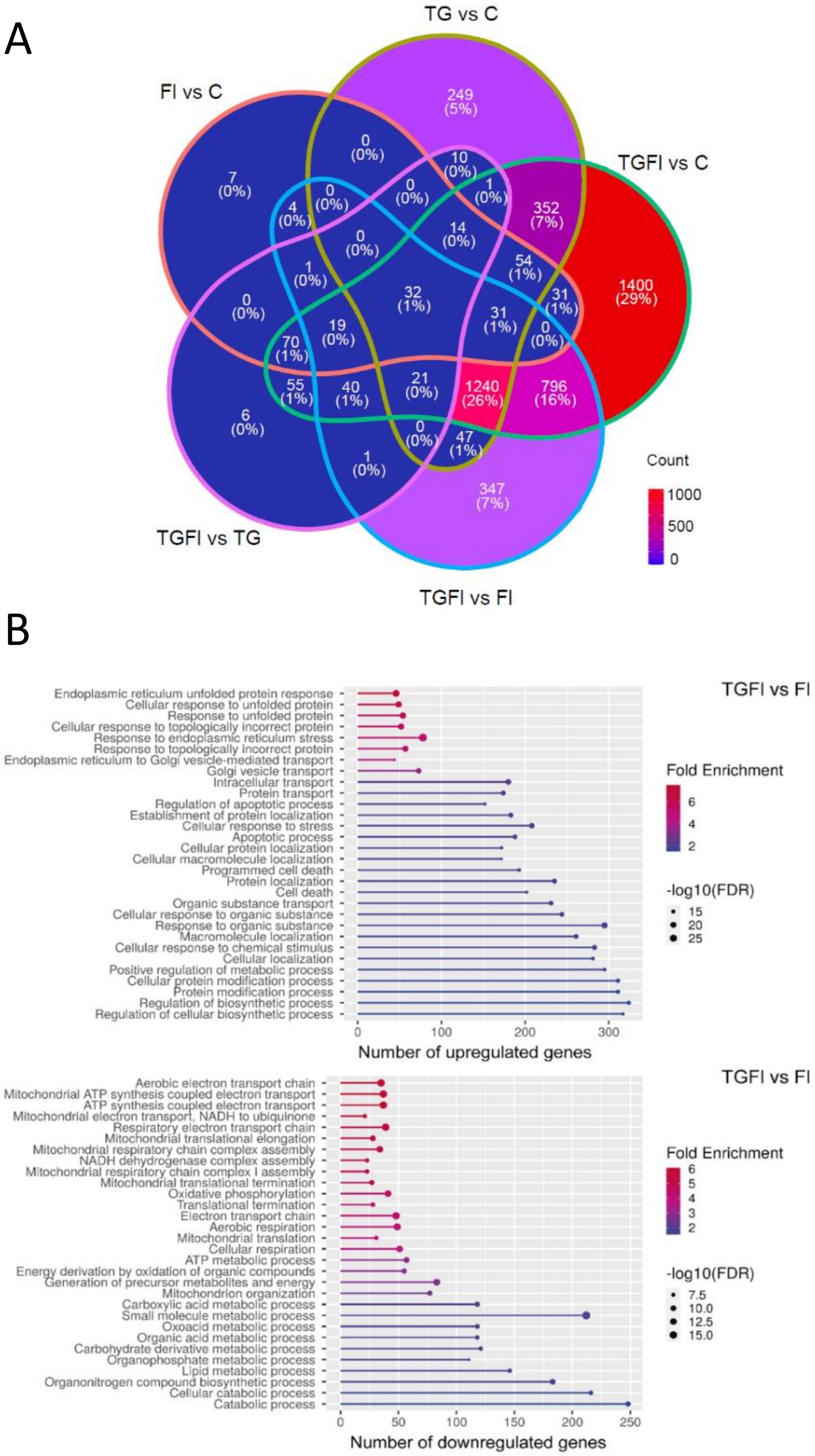

Supplementary Figure 3

TOP CANONICAL PATHWAYS		
**Fl vs Control**	**TG vs Control**	**TG+Fl vs Control**
IL-6 signaling<br>HMBG1 signaling<br>Acute Phase signaling<br>Rho-family GTPases	IL-6 signaling<br>HMBG1 signaling<br>Colorectal cancer metastasis signaling<br>IL-8 signaling<br>Glycolysis I*<br>LPS/IL-1 mediated inhibition of RXR*<br>FXR/RXR activation*<br>Oxidative phosphorylation*<br>Mitochondrial disfunction*<br><br>*(all these pathways downregulated)	IL-6 signaling<br>IL-17 signaling<br>NF-κB signaling<br>HMBG1 signaling<br>B cell receptor response signaling<br>Unfolded protein response<br>Colorectal cancer metastasis signaling<br>Glutathione-mediated Detoxification

TOP UPREGULATED MOLECULES		
**Fl vs Control**	**TG vs Control**	**TG+Fl vs Control**
CXCL1<br>IL17C<br>CXCL8<br>CXCL3<br>CXCL10<br>BIRC3<br>SPRR2A<br>CSF3<br>CC4L1/CCL4L2<br>CXCL2	SPRR2A<br>DRD5<br>KLHDC7B<br>C11orf96<br>IGFBP1<br>POU5F2<br>CCL18<br>VLDR-AS1<br>CES5A	CXCL8<br>SPRR2A<br>C11orf96<br>CXCL1<br>CXCL3<br>CSF3<br>CC4L1/CCL4L2<br>IL17C<br>BIRC3<br>CXCL2

TOP DOWNREGULATED MOLECULES		
**Fl vs Control**	**TG vs Control**	**TG+Fl vs Control**
MTM1<br>RNBP3L<br>SLC22A16<br>HS3ST4<br>KRTAP3-2<br>ENKUR<br>POFUT1	ENKUR<br>SAA4<br>S100G<br>CYP8B1<br>MS4A10<br>PCDH9<br>BTNL3<br>ARG1	MSMB<br>S100A1<br>PTGS<br>TRAF3PI3<br>SCML4<br>SXYD2<br>SLC22A2<br>SAA4<br>RANBP3L

Supplementary Figure 4

A

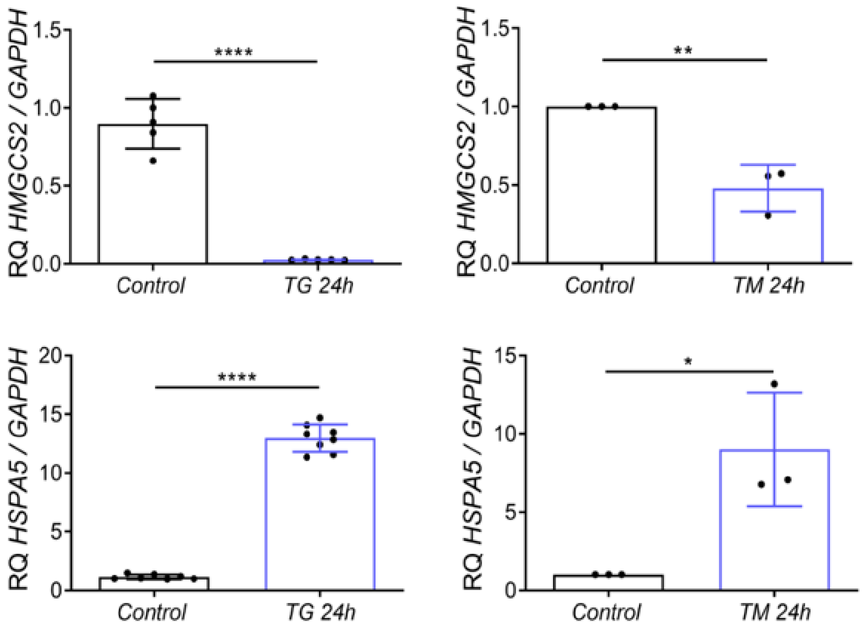

B

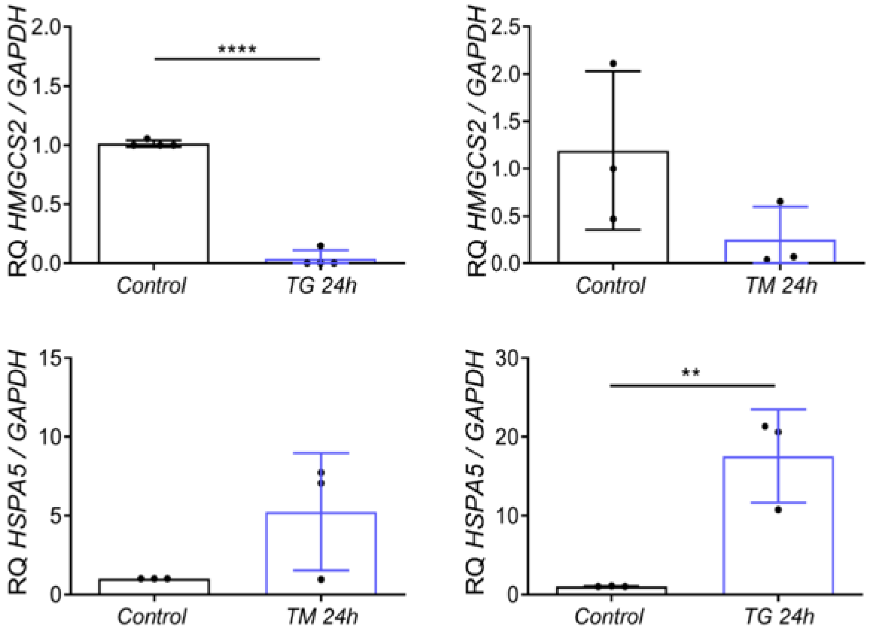

Supplementary Figure 5

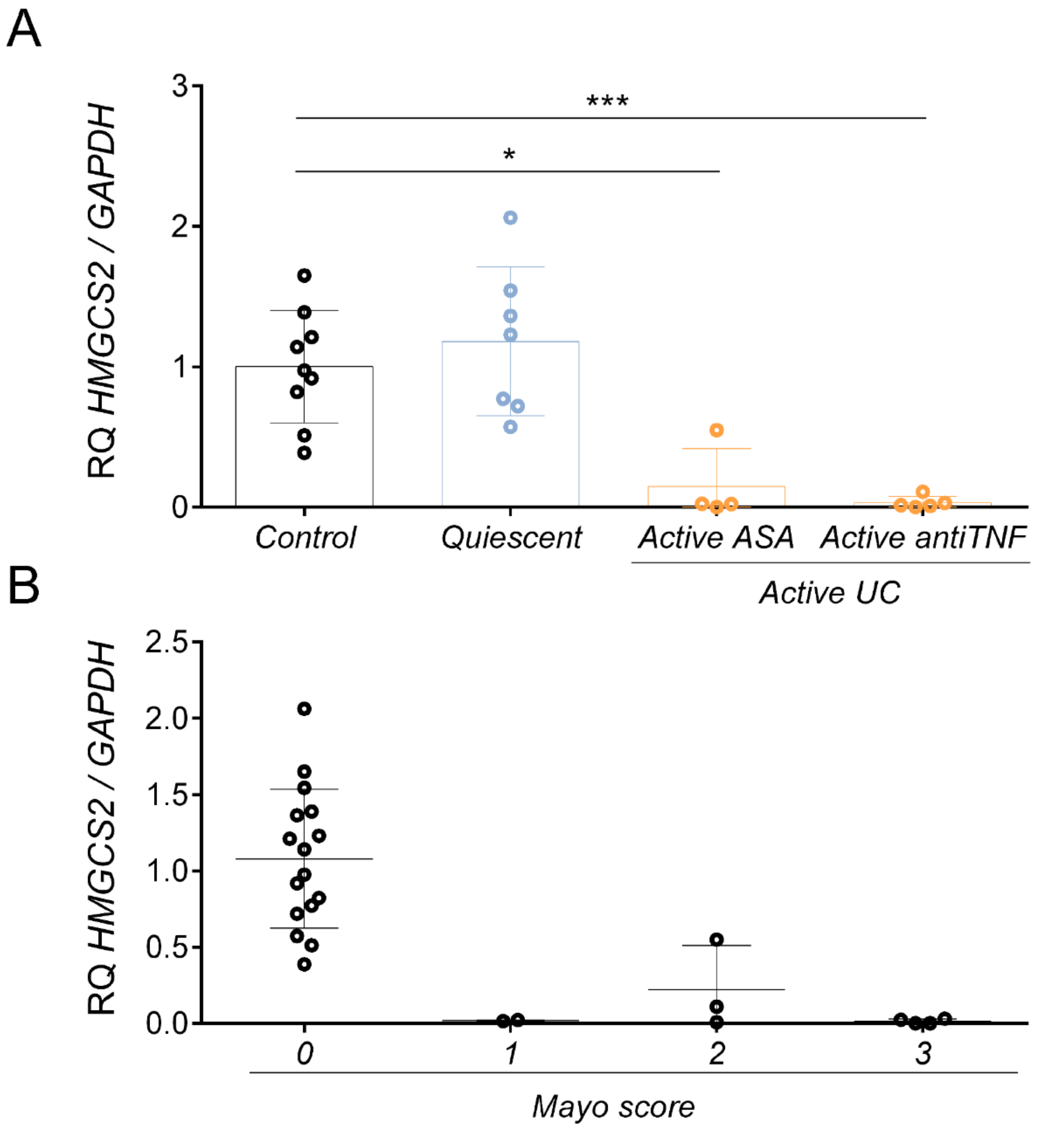

Supplementary Figure 6

A

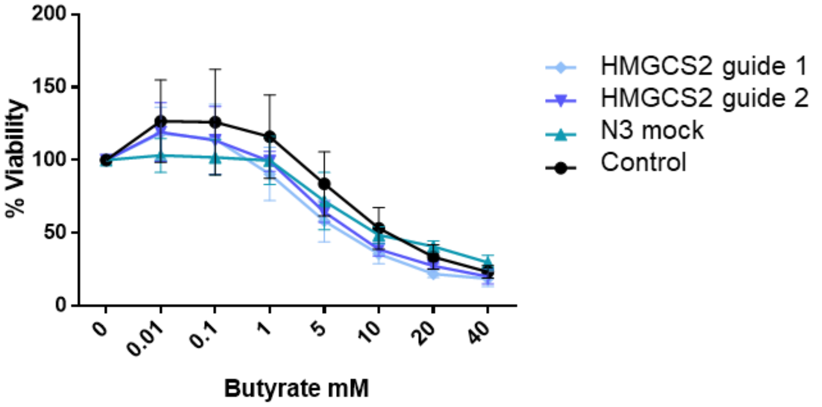

B

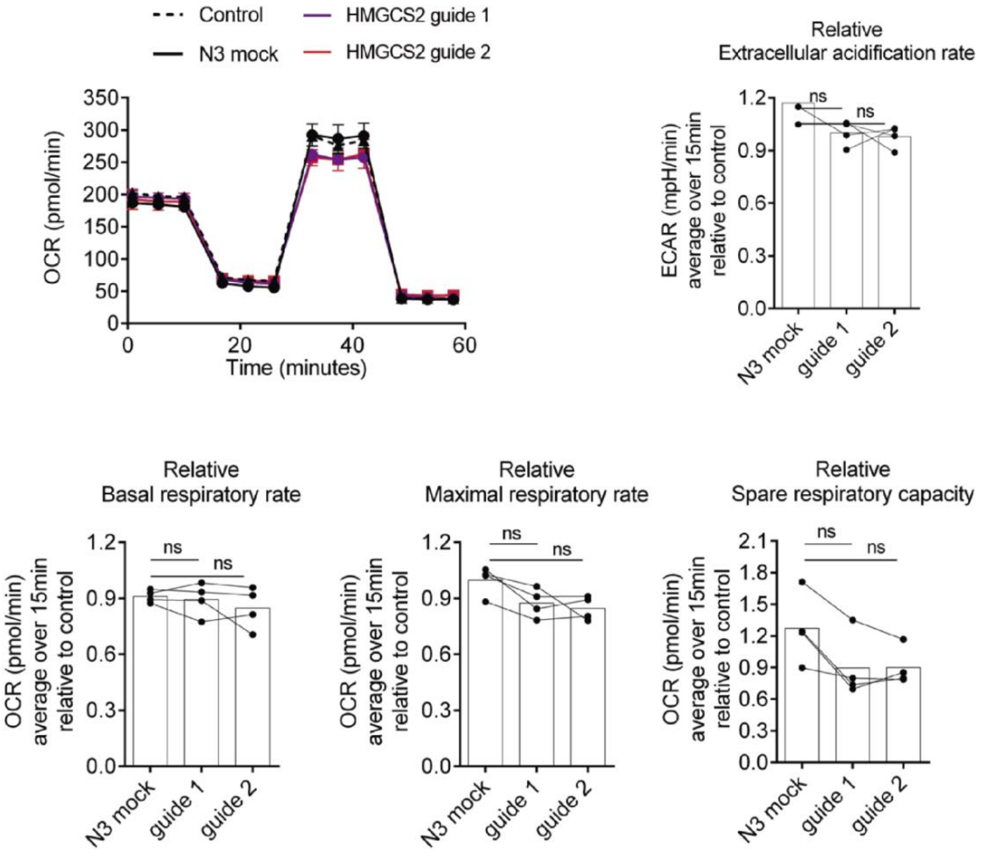

SUPPLEMENTARY FIGURE LEGENDS

Supplementary Figure 1. Activation of the UPR in Caco-2 cell line after treatment with flagellin (Fl), thapsigargin (TG) and the combination of both treatments (TGFl) at different time points. Representative immunoblots showing **e** the effect on BIP, P-eIF2α, eIF2α and ATF4 expression, compared to tubulin. **B)** g the effect of each treatment on XBP1 splicing using actin as a loading control. XBP1 U, unspliced form. XBP1S, spliced form. **C)** the effect of TG treatment on P-eIF2α, , ATF4 and ATF6 expression.

Supplementary Figure 2. Transcriptional profile analysis of Caco-2 cell line upon inflammatory signals and ER stress induction.
A) Venn diagram representing all differentially expressed genes (DEGs) between groups, with a significance level of 0.01. Different treatments are represented as C (non-treated cells), Fl (treated with flagellin 1 µg / mL for 2 hours), TG (treated with TG at 1 µM for 5 hours), or TGFl (treated with 1 µM TG for 5 hours, and with 1 µg / mL Fl for additional 2 hours). Each compartment shows the number and percentage of deregulated genes between the compared groups. **B)** GO enrichment analysis representing the 30 top upregulated and downregulated pathways based on DEGs by biological process (BP). Selected genes were those upregulated and downregulated between TGFl and FL treatments, using a p-value cut-off of 0.01. The color represents the Fold enrichment, the length of each bar represents the number of upregulated genes associated to that pathway and the size of the circle represents the False Discovery Rate (FDR).

Supplementary Figure 3. Ingenuity Pathway Analysis (IPA) of the human colorectal adenocarcinoma cell line Caco-2 treated with thapsigargin (TG), a chemical inducer of ER stress, flagellin (Fl), a proinflammatory stimulus and the combination of TG plus Fl (TGFl). Top canonical molecules and pathways deregulated were calculated for the three different treatments upon the highest p-values obtained in the IPA. Top up- and down-regulated molecules based on Fold change were calculated using a cut-off value between -1.5 and 1.5.

Supplementary Figure 4. HMGCS2 is downregulated by ER stress in two different colorectal cell lines (Caco-2 and HT29) after exposure to different ER Stress inducers (TG and TM). A) Caco-2 cells**. B)** HT-29 cells. The relative abundance of *HMGCS2* or *HSPA5* mRNAs was determined by qPCR using GAPDH as the housekeeping gene. Cells were treated with 1μM TG or 2μgr/ml TM for 24 hours and compared to non-treated cells (Control). Data are represented as the mean ± SEM. Unpaired t test was used. *P<0.05, **P<0.01, ***P<0.001, ****P<0.0001.

Supplementary Figure 5. *HMGCS2* is downregulated in colonic samples from patients with active UC regardless of their treatment or the severity score of the sample. Relative mRNA expression levels of *HMGCS2* normalized to *GAPDH* in 25 colonic biopsies of healthy donors and UC patients with quiescent or active disease, classified according to (A) the treatment they were receiving at the moment of biopsy or (B) the Mayo score assigned by the pathologist. Mann-Whitney U test was used. Data are represented as the mean ± SEM *P<0.05, **P<0.01, ***P<0.001, ****P<0.0001.

Supplementary Figure 6. Viability and bioenergetic analyses of Control Caco-2 (control parental cells and Mock Caco-2) and HMGCS2-deficient Caco-2 cell lines. A) Viability of Caco-2 cells asses by MTT in the presence of different concentrations of butyrate. Data represent the mean ± SEM of three independent experiments**. B)** Bioenergetic analysis of Caco-2 cells cultures in complete medium in response to different metabolic inhibitors. The results are normalized to control parental Caco-2 cell values. Oxigen consumption rates (OCR) and Extracellular acidification rate (ECAR) were measured three times over 15 minutes as a readout for the mitochondrial respiration and glycolysis respectively. Data shows a representative respiration plot (up left) or the mean and paired individual data points of 4 independent experiments. One-Way ANOVA corrected with Tukey's multiple comparisons test was used. ns not significant (P>0.05), *P<0.05 **P<0.01, ***P<0.001, ****P<0.0001.

Supplementary Table 1. Primers used in gene expression studies

Gene	Primer Forward (5'- 3')	Primer Reverse (5'- 3')
HSPA5	agctgtagcgtatggtgctg	aaggggacatacatcaagcagt
CXCL8	agacagcagagcacacaagc	aggaaggctgccaagagag
CXCL2	cccatggttaagaaaatcatcg	cttcaggaacagccaccaat
SPRR2A	aacccctggtacctgagca	cttgcactgctgctgttgat
MMP3	caaaacatatttctttgtagaggacaa	ttcagctatttgcttgggaaa
MMP7	gctgacatcatgattggcttt	tctcctccgagacctgtcc
ULBP1	tgggggattgtaagatgtgg	gccagagagggtggttttg
ULBP2	ccgctaccaagatccttctg	ggatgacggtgatgtcatagc
ULBP3	caggcttagctcaacccaaa	gcttcttgatatcaccttccactc
ANXA1	cagtaagcatgacatgaacaaagtt	gaaagctggtttgcttgtgg
DUSP6	cgactggaacgagaatacgg	ttggaacttactgaagccacct
HMGCS2	cagcaagtttcttttcatttcg	gtgctggacaccaacttgtc
GAPDH	ctctgctcctcctgttcgac	acgaccaaatccgttgactc

Supplementary Table 2. Primary antibodies used for the detection of proteins by Western Blot

Antigen	Dilution	Reference	Company
BiP	1.1000	C50B12	Cell Signaling
ATF4	1.500	D4b8	Cell Signaling
ATF6	1.500	MAB71527	R&D
Phospho-eIF2α (Ser51)	1.1000	9721	Cell Signaling
eIF2α	1.1000	9722	Cell Signaling
HMGCS2	1.1000	Ab137043	Cell Signaling
GAPDH	1.5000	sc-47724	Santa Cruz Biotechnology

Supplementary Table 3. Secondary antibodies used for the detection of proteins by Western Blot

Antigen	Dilution	Reference	Company
Donkey Anti-Mouse IgG IRDye 800 CW	1.10000	926-32212	LI-COR Biosciences
Goat Anti-Rabbit IgG IRDye 680 RD	1.10000	926-68071	LI-COR Biosciences
Goat Anti-Rabbit IgG (H+L) HRP conjugate	1.5000	AP307P	Merck Millipore

Supplementary Table 4. Primary antibodies used for the detection of proteins by Immunohistochemistry

Antigen	Dilution	Reference	Company
HMGCS2	1/50	Ab137043	Cell Signaling
Negative control Rabbit Immunoglobulin fraction	10 µg/ml	X0903	Dako (Agilent)
